# High-resolution mapping of age- and gender-specific risk of *Clonorchis sinensis* infection risk in Guangdong, China: a geostatistical modeling study

**DOI:** 10.1186/s13071-024-06166-z

**Published:** 2024-02-16

**Authors:** Si-Yue Huang, Jing-Diao Chen, Qing-Sheng Zeng, Ying-Si Lai

**Affiliations:** 1 Department of Medical Statistics, School of Public Health, Sun Yat-Sen University, Guangzhou, Guangdong Province People’s Republic of China; 2 Guangdong Provincial Center for Disease Control and Prevention, Guangzhou, Guangdong Province People’s Republic of China; 3 Xinhui District Center for Disease Control and Prevention, Jiangmen, Guangdong Province People’s Republic of China; 4 Sun Yat-Sen Global Health Institute, Sun Yat-Sen University, Guangzhou, Guangdong Province People’s Republic of China

**Keywords:** Clonorchiasis, High-resolution mapping, Intervention, Guangdong Province

## Abstract

**Background:**

The latest national survey on the distribution of human parasites in China demonstrated that Guangdong was among the endemic provinces with the highest *Clonorchis sinensis* infection rates. High-resolution, age- and gender-specific risk maps of the temporal and spatial distributions are essential for the targeted control work.

**Methods:**

Disease data on the prevalence of *C.* *sinensis* infection from 1990 onwards, either age- and gender-specific or aggregated across age and gender, were collected through systematic review and four large-scale surveys in Guangdong Province. Environmental and socioeconomic variables were obtained from open-access databases and employed as potential predictors. A Bayesian geostatistical model was developed to estimate the *C.* *sinensis* infection risk at high spatial resolution.

**Results:**

The final dataset included 606 surveys at 463 unique locations for *C.* *sinensis* infection. Our findings suggested that following an initial increase and stabilization, the overall population-adjusted prevalence had declined to 2.2% (95% Bayesian credible interval: 1.7–3.0%) in the period of 2015 onwards. From 2015 onwards, moderate and high infection risks were found in the northern regions (e.g. Heyuan and Shaoguan cities) and the southern Pearl River Delta (e.g. Foshan, Zhongshan, Zhuhai and Jiangmen cities), respectively. Age- and gender-specific risk maps revealed that males had a higher infection risk than females, and the infection risk was higher in adults compared to children.

**Conclusions:**

Our high-resolution risk maps of *C.* *sinensis* infection in Guangdong Province identified the spatial, temporal, age and gender heterogeneities, which can provide useful information assisting tailored control strategies.

**Graphical Abstract:**

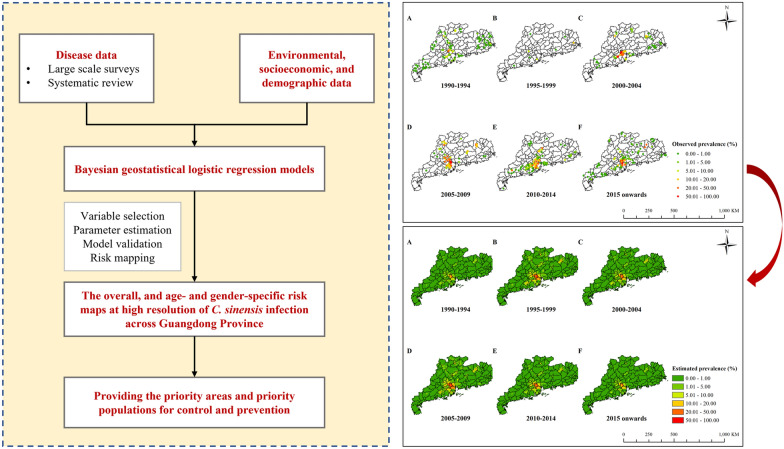

**Supplementary Information:**

The online version contains supplementary material available at 10.1186/s13071-024-06166-z.

## Background

Clonorchiasis is an important foodborne parasitosis caused by infection with *Clonorchis sinensis* (*C.* *sinensis*). The parasite has a complex life cycle, involving freshwater snails as the first intermediate host and freshwater fish and occasionally shrimps as the second intermediate host. Human or carnivorous mammals, as the definitive host, are infected with *C.* *sinensis* mainly through the consumption of raw or undercooked freshwater fish carrying metacercariae [1–3]. The mild infections often have nonspecific symptoms [4, 5], whereas severe infections may lead to various complications, including cholelithiasis, cholecystitis, cholangitis and cholangiocarcinoma [6, 7]. Furthermore, *C. sinensis* was identified by the International Agency for Research on Cancer (IARC) as a group 1 carcinogen of humans [8]. Approximately 15 million people were estimated to be infected with *C.* *sinensis*, the majority of whom lived in East Asia (i.e. China, the Republic of Korea and northern Vietnam) [9, 10]. Particularly, China accounted for the largest share (85%) with an estimated 13 million people infected [11, 12]. There were two major endemic regions in China: the southeastern region (including Guangxi and Guangdong provinces) and the northeastern region (including Heilongjiang and Jilin provinces) [13, 14].

Guangdong Province, located in southeast China, covered by dense river networks, is abundant in aquatic products [15, 16]. In certain areas of the province, residents have the deeply rooted habit of eating raw freshwater fish [17, 18]. Up to now, several large-scale surveys have been carried out in Guangdong. The first and the second national surveys, conducted in 1988–1992 and 2001–2004, respectively, revealed that Guangdong had the highest prevalence (1.8% and 5.4%) of *C.* *sinensis* infection across China [19, 20]. In 2010, a large provincial survey observed an increased prevalence of 6.2% [21]. This number dropped to 4.2% in the third national survey (2015–2016), the latest one, ranking second among all provinces in the country [22]. Results obtained from the above four surveys showed a downward trend of prevalence from 2010 onwards but a significant heterogeneity in space. Particularly, moderate-to-high prevalence was found in the southern Pearl River Delta part and the northern areas [22]. The time intervals between the above surveys were relatively large, during which small surveys were conducted across different locations in the province. However, it is difficult to obtain the spatial-temporal risk in different areas only based on the simple statistical description of historical survey data.

High-resolution risk maps, showing the temporal and spatial distribution of disease risk, are particularly important in guiding control strategies and intervention plans [23, 24]. Bayesian geostatistical modeling, as a flexible and robust inferential approach to producing high-resolution disease risk maps [25], has been widely used in multifarious studies on foodborne parasitosis in different countries or regions [26–30]. Based on this method, Lai et al. produced a risk map of *C.* *sinensis* infection in China, including the high-resolution spatial distribution of *C.* *sinensis* infection in Guangdong [26]. However, the above study, based on survey data between 2000 and 2015, just roughly separated the study period into two segments and thus could not reflect either the detailed temporal changes or the current status of the disease. Besides, as data were aggregated regardless of age and gender, age- and gender-specific infection heterogeneities were not considered. To address the above limitations, in this study, we aimed (i) to provide age- and gender-specific risk maps and (ii) to evaluate the current status and the temporal changes in *C.* *sinensis* infection risk over 30 years in Guangdong, thus providing important references for control and prevention of the disease in the province.

## Methods

### Data source

#### Disease data

As most of the surveys on clonorchiasis in Guangdong have been carried out since the first national survey around 1990, we set our study period from 1990 onwards. Geo-referenced data on clonorchiasis from the four large-scale surveys were aggregated as the number of examined and number of positive individuals in two genders (male and female) and seven age groups (0–9, 10–19, 20–29, 30–39, 40–49, 50–59 and ≥ 60 years old), provided by Guangdong Provincial Center for Disease Control and Prevention (Guangdong CDC). Additionally, we did a systematic review to collect relevant papers, extending the study time of Lai’s work [26]. In Lai’s study, a systematic review was undertaken in PubMed, ISI Web of Science, China National Knowledge Internet (CNKI) and Wanfang Data from January 1, 2000, until January 10, 2016 [26]. Our extension review identified studies from January 1, 1990, to December 31, 1999, and from January 10, 2016, to March 17, 2023. We followed Lai’s study [26] to set the search terms and the criteria for inclusion, exclusion and extraction of data. Finally, we combined all available disease data from the above sources for subsequent analysis. Geographical coordinates of survey locations were obtained via Google Maps (https://www.google.com).

#### Environmental, socioeconomic and demographic data

Environmental, socioeconomic and demographic data were obtained from different open-access data sources (with details in Additional file 1: Table S1). Land surface temperature (LST) in the daytime and at night and normalized difference vegetation index (NDVI) were averaged yearly. According to between-class similarities, we reclassified land cover data into five categories: (i) forest; (ii) shrublands and grass; (iii) wet areas; (iv) croplands; (v) urban. All data were converted to raster files with 5 × 5 km^2^ spatial resolution, identical to the resolution of risk maps of our study.

### Statistical analysis

As most of the surveys used Kato-Katz as the diagnostic method, to ensure consistency in the diagnostic technique, we only kept prevalence data diagnosed by this method. A sample size *n* = 50 was assigned to surveys where only prevalence was reported. The year of publication minus 3 was assigned to the survey with the survey date missing. The survey period was equally divided into six categories: 1990–1994, 1995–1999, 2000–2004, 2005–2009, 2010–2014 and from 2015 onwards. Data obtained from the literature were community-based, aggregated across age and gender, but data provided by Guangdong CDC were age group- and gender-specific; we introduced an age-gender-specific variable containing 15 age-gender groups to comprehensively analyze all available data reflecting age and gender heterogeneity: (i) surveys aggregated across age and gender, (ii) females aged < 10 years, (iii) males aged < 10 years, (iv) females aged 10–19 years, (v) males aged 10–19 years, (vi) females aged 20–29 years, (vii) males aged 20–29 years, (viii) females aged 30–39 years, (ix) males aged 30–39 years, (x) females aged 40–49 years, (xi) males aged 40–49 years, (xii) females aged 50–59 years, (xiii) males aged 50–59 years, (xiv) females aged ≥ 60 years and (xv) males aged ≥ 60 years. Particularly, the first group referred to the data obtained from the literature that aggregated across age and gender. Categorical variables were transformed into dummy forms, and continuous variables were standardized to mean 0 and standard deviation 1. As for any pair of continuous variables with Pearson’s correlation coefficient > 0.7, we dropped the one with lower quality to avoid collinearity.

Bayesian geostatistical logistic regression models with spatially specific random effects were applied to analyze the survey data with potential predictors. Here, ${Y}_{{i}^{k}},{ n}_{{i}^{k}},{ p}_{{i}^{k}}$ were respectively defined as the number of positive individuals, number of examined people, and prevalence of infection in age-gender group *k* (*k* = 1, 2, 3, …, 15, representing the corresponding group of the age-gender specific variable, respectively) at survey location *i* (*i* = 1, 2, 3, …,* l*). We assumed that ${Y}_{{i}^{k}}$ arose from a binomial distribution ${Y}_{{i}^{k}}\sim Bin({p}_{{i}^{k}}, {n}_{{i}^{k}})$, where $logit\left({p}_{{i}^{k}}\right)={\beta }_{0}+{\sum }_{m=1}{\beta }_{m}\times {X}_{i}^{(m)}+{\vartheta }_{i}$. ${\beta }_{0}$ was the intercept, and ${\beta }_{m}$ was the regression coefficient of the ${mth}$ covariate ${X}_{i}^{(m)}$. The spatial random effect $\boldsymbol{\vartheta }$ was assumed to follow a zero-mean Gaussian distribution with a Matérn covariance function, that is $Cov({\vartheta }_{i}, {\vartheta }_{j})=\frac{{\sigma }_{sp}^{2}}{{2}^{v-1}\Gamma (v)}{(\kappa {d}_{ij})}^{v}{K}_{v}(\kappa {d}_{ij})$. Here, ${\sigma }_{sp}^{2}$ was spatial variance, expressed as $1/({4\pi \kappa }^{2v}{\tau }_{sp}^{2})$. ${d}_{ij}$ was denoted the Euclidean distance between locations *i* and *j*, and *κ* was a scaling parameter. ${K}_{v}$ was the modified Bessel function of the second kind, where $v=1$ was regarded as a smoothing parameter. The spatial range, $r=\sqrt{8v}/\kappa$, denoted the distance at which spatial correlation becomes negligible (< 0.1).

The model was fitted through the integrated nested Laplace approximations (INLA) approach in a Bayesian framework, using the INLA package in R. We adopted less informative priors as follows: normal prior distributions for the intercept and regression coefficients as ${\beta }_{0},{\beta }_{m}\sim N (0, 1000)$ and log normal distributions for hyperparameters ${\tau }_{sp}$ and $\kappa$ as ${\text{log}}({\tau }_{sp})\sim \mathrm{log }normal(\mathrm{0,100})$ and ${\text{log}}(\kappa )\sim \mathrm{log }normal(\mathrm{0,100})$, respectively.

Moreover, to obtain the best set of predictors for a parsimonious model, a variable selection process was adopted. First, to identify the best functional form of continuous variables, continuous ones were divided into three-level categorical ones based on a preliminary exploration for potential non-linear outcome predictor relationships. We respectively developed two univariate Bayesian geostatistical models for the continuous or categorical form of each continuous predictor and chose the form with the minimum log score as the best functional form. Second, the backward elimination approach was used to identify the best set of fixed effect covariates for the final model [31]. Additionally, based on previous studies, survey period, age and gender were identified as the influencing factors for *C.* *sinensis* infection [32–34]; hence, the corresponding variables were kept in the models during the whole variable selection process.

For model validation, all observed locations (*N*) were randomly divided into two sets, with 80% of locations as a training set and the remaining 20% as a validation set. We calculated the following indicators to assess model performance: mean error ($ME=\frac{1}{N}\times \sum_{i=1}({\pi }_{i}-{\widehat{\pi }}_{i})$), mean absolute error $\left( {MAE = \frac{1}{N} \times \mathop \sum \limits_{i = 1} \left| {\pi_{i} - \hat{\pi }_{i} } \right|} \right)$, the coverage of observations within 95% Bayesian credible intervals (BCIs) of posterior distribution of estimated prevalence and the area under the receiver-operating characteristic (ROC) curve (AUC). Here, ${\pi }_{i}$ and ${\widehat{\pi }}_{i}$ were denoted as the observed and estimated prevalence at location *i*.

Age- and gender-specific estimates of *C.* *sinensis* infection risk were obtained using Bayesian kriging at each pixel of a regular grid of 5 × 5 km^2^ spatial resolution across Guangdong Province. We calculated the overall estimated prevalence and the number of infected individuals at both provincial and city levels, based on pixel-level age- and gender-specific estimated prevalence weighted by population density. To validate the rationality of the grouping method of age and gender, we compared the overall prevalence estimated based on age- and gender-specific prevalence and that based on surveys aggregated across age and gender.

All statistical analyses were conducted using R software (version 4.0.3), and the high-resolution risk maps were depicted using ArcGIS (version 10.2).

## Results

### Data summaries

**Fig. 1:**
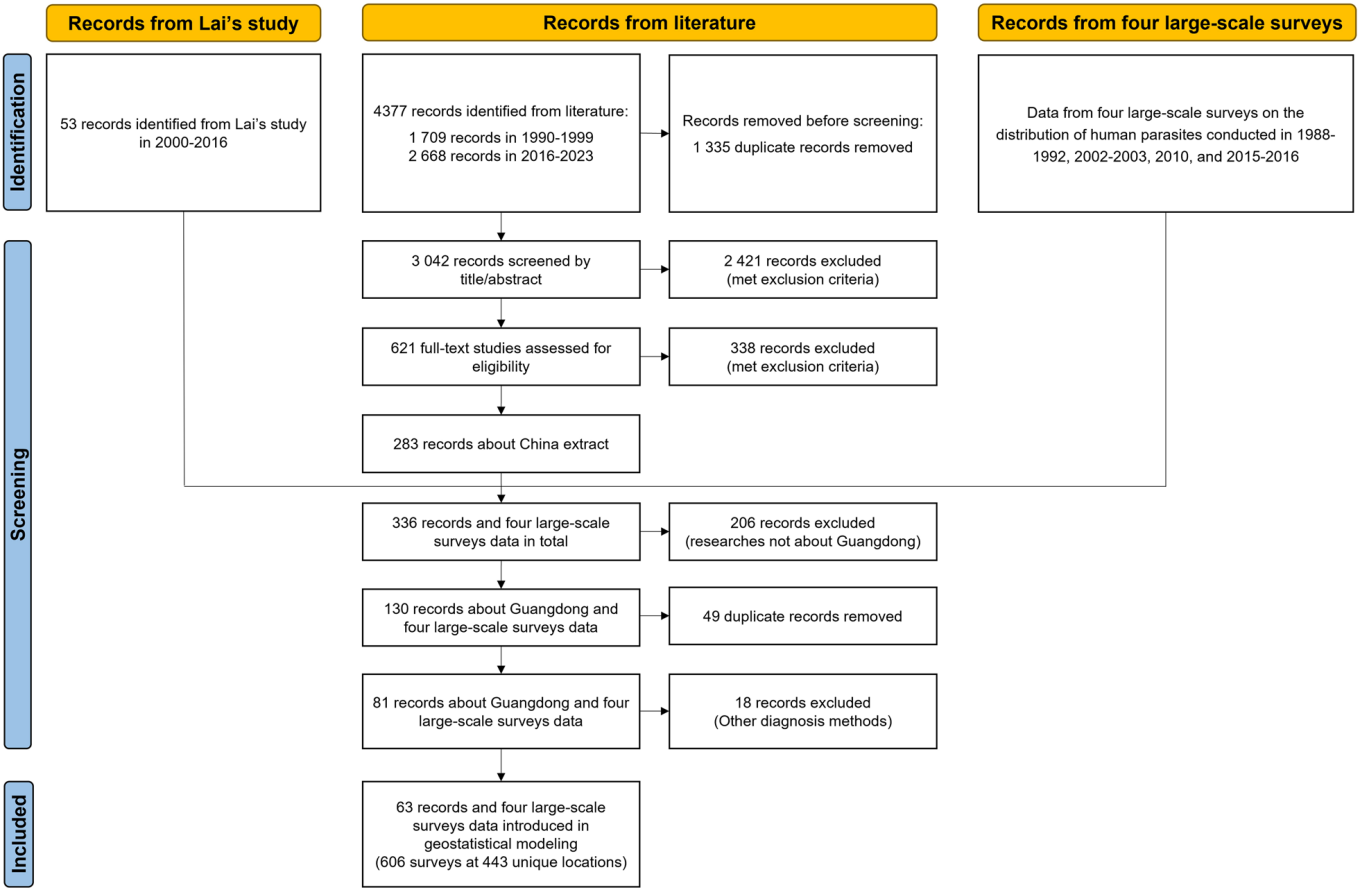
Data selection flow chart

**Fig. 2:**
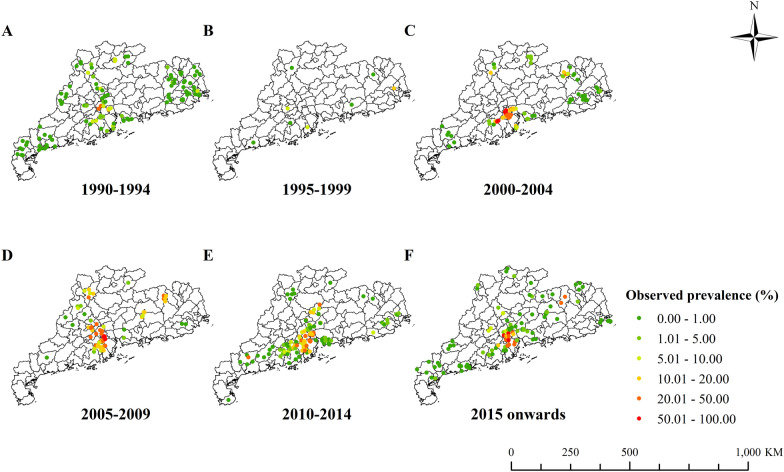
Survey locations and observed prevalence in Guangdong. **A** 1990–1994, **B** 1995–1999, **C** 2000–2004, **D** 2005–2009, **E** 2010–2014 and **F** 2015 onwards

We identified 4377 records through a literature search, while 53 records stemmed from Lai’s study, and the four large-scale surveys' data were provided by Guangdong CDC (Fig. 1). According to the inclusion and exclusion criteria, the final dataset included 606 surveys on *C.* *sinensis* infections at 443 unique locations from 1990 to 2023. The summary of survey data is shown in Additional file 1: Table S2, while survey locations with the observed prevalence in each period are presented in Fig. 2.

### Variable selection, geostatistical modeling and model validation

After variable selection, we included seven covariates for the final Bayesian geostatistical model. The posterior summaries of model parameters are shown in Table 1. Compared to the infection risk in the 1990–1994 period, higher risk was shown in the following periods, until the period 2015 onwards, when a lower risk was detected. There were significant differences in infection risk between age and gender groups. Females had a lower risk than males in each age group, and the infection risk increased significantly with age until 50–59 years old, where a slightly downward trend was shown. Compared to surveys aggregated across age and gender, all female age groups had a lower infection risk, while males > 30 years old showed a higher risk. Regarding land cover, there was a higher *C.* *sinensis* infection risk for people living in urban areas compared to people living in shrub and grass areas. Conversely, people living in cropland areas had a lower infection risk than those living in shrub and grass areas. A positive association was found for the infection risk of *C.* *sinensis* with LST at night and night light, while a negative association was found with precipitation.

**Table 1:** Posterior summaries of model parameters

Variable	Estimated median (95% BCI)	*OR*	Posterior probability of *OR* > 1
Year			
1990–1994	Ref	Ref	–
1995–1999	0.98 (−0.32, 2.27)	2.68 (0.72, 9.69)	0.95
2000–2004	0.43 (0.27, 0.59)^a^	1.54 (1.31, 1.80)	> 99.99
2005–2009	1.01 (0.81, 1.22)^a^	2.75 (2.24, 3.39)	> 99.99
2010–2014	0.40 (0.21, 0.59)^a^	1.49 (1.24, 1.80)	> 99.99
≥ 2015	−0.44 (−0.63, −0.24)^a^	0.64 (0.53, 0.78)	< 0.01
Age-gender group (years)			
Surveys across age and gender	Ref	Ref	–
Female (0–9)	−2.15 (−2.40, −1.91)^a^	0.12 (0.09, 0.15)	< 0.01
Female (10–19)	−1.35 (−1.53, −1.18)^a^	0.26 (0.22, 0.31)	< 0.01
Female (20–29)	−0.79 (−0.95, −0.63)^a^	0.45 (0.39, 0.53)	< 0.01
Female (30–39)	−0.58 (−0.72, −0.44)^a^	0.56 (0.49, 0.64)	< 0.01
Female (40–49)	−0.63 (−0.78, −0.48)^a^	0.53 (0.46, 0.62)	< 0.01
Female (50–59)	−0.31 (−0.46, −0.16)^a^	0.73 (0.63, 0.85)	< 0.01
Female (≥ 60)	−0.49 (−0.65, −0.33)^a^	0.61 (0.52, 0.72)	< 0.01
Male (0–9)	−1.94 (−2.15, −1.75)^a^	0.14 (0.12, 0.17)	< 0.01
Male (10–19)	−1.11 (−1.27, −0.95)^a^	0.33 (0.28, 0.39)	< 0.01
Male (20–29)	−0.07 (−0.22, 0.08)	0.93 (0.80, 1.08)	0.18
Male (30–39)	0.40 (0.27, 0.53)^a^	1.49 (1.30, 1.70)	> 99.99
Male (40–49)	0.57 (0.43, 0.70)^a^	1.76 (1.54, 2.01)	> 99.99
Male (50–59)	0.65 (0.50, 0.79)^a^	1.91 (1.66, 2.20)	> 99.99
Male (≥ 60)	0.46 (0.31, 0.61)^a^	1.59 (1.36, 1.84)	> 99.99
LST at night (°C)	0.21 (0.11, 0.30)^a^	1.23 (1.12, 1.35)	> 99.99
LST in the daytime (°C)			
< 24.48	Ref	Ref	–
24.48–28.47	−0.24 (−0.36, −0.12)^a^	0.78 (0.70, 0.88)	< 0.01
≥ 28.47	0.20 (−0.03, 0.43)	1.22 (0.97, 1.54)	0.96
Precipitation (mm)			
< 16337.64	Ref	Ref	–
16337.64–18856.80	−0.29 (−0.37, −0.21)^a^	0.75 (0.69, 0.81)	< 0.01
≥ 18856.80	−0.23 (−0.31, −0.15)^a^	0.79 (0.73, 0.86)	< 0.01
Night light	0.47 (0.35, 0.59)^a^	1.60 (1.43, 1.80)	> 99.99
Land cover			
Shrub and grass	Ref	Ref	–
Forest	0.45 (−1.30, 2.18)	1.58 (0.27, 8.82)	0.69
Urban	0.24 (0.07, 0.42)^a^	1.27 (1.07, 1.52)	> 99.99
Wet area	0.06 (−0.18, 0.30)	1.06 (0.84, 1.35)	0.69
Crop	−1.95 (−3.03, −1.01)^a^	0.14 (0.05, 0.36)	< 0.01
Spatial range (km)	19.58 (16.02, 23.95)	–	–
*σ*_*sp*_	9.12 (7.07, 11.81)	–	–

^a^Significant effect identified by not including zero in the 95% BCI of posterior distribution of the corresponding coefficient

The final model was able to correctly estimate (within the 95% BCIs) 89.7% of the locations. The ME and MAE were 0.8% and 3.8%, respectively, suggesting that our model might slightly underestimate the infection risk (Additional file 1: Fig. S1). Additionally, there was no significant difference between the overall population-adjusted estimated prevalence based on age- and gender-specific prevalence (2.2%, 95% BCI: 1.7%–3.0%, in 2015 onwards) and that based on surveys aggregated across age and gender (2.7%, 95% BCI: 2.1%−3.7%, in 2015 onwards), suggesting that the grouping method of age and gender was rational.

### Risk maps and estimated number of people infected

**Fig. 3:**
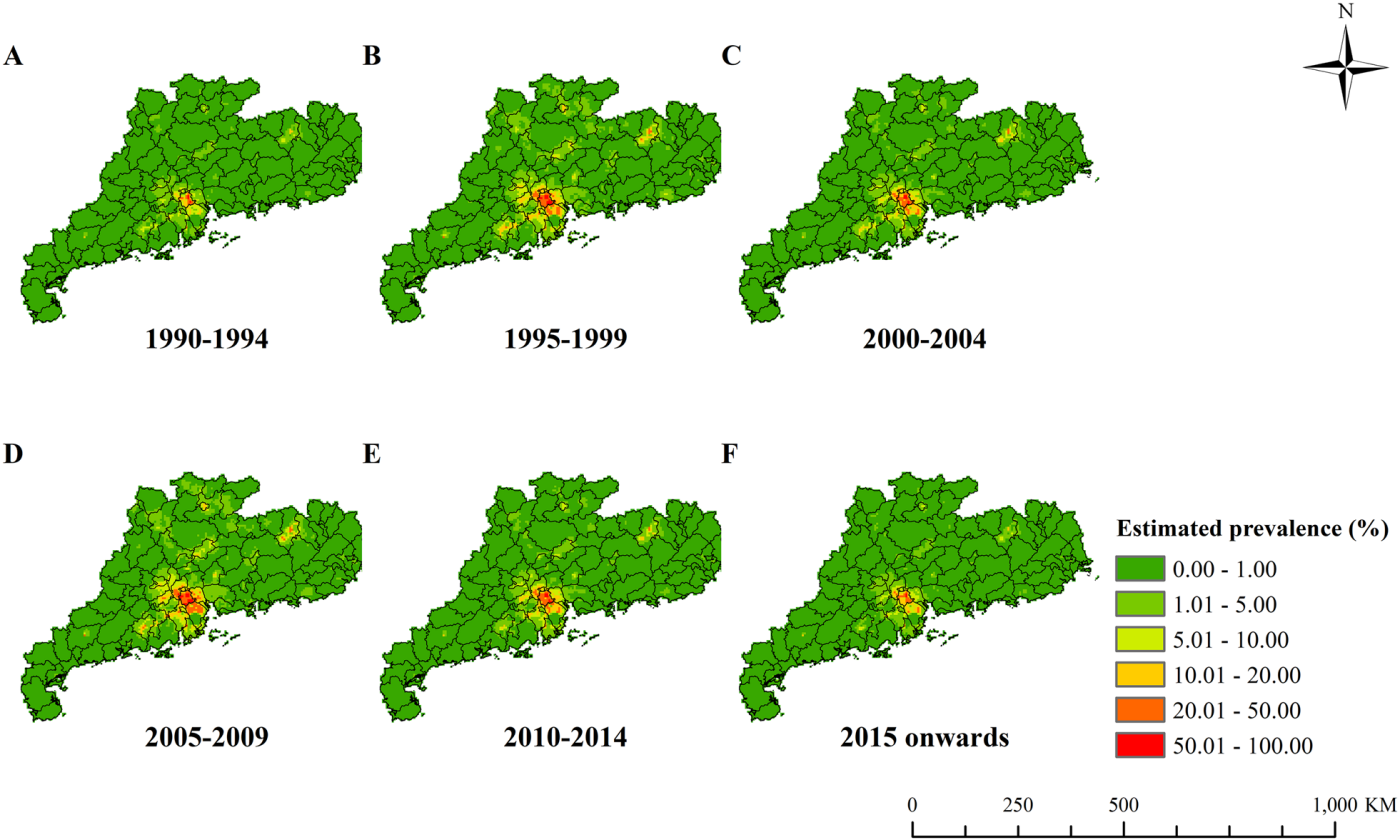
The geographical distribution of *Clonorchis* *sinensis* infection risk in Guangdong Province in different time periods. **A** 1990–1994, **B** 1995–1999, **C** 2000–2004, **D** 2005–2009, **E** 2010–2014 and **F** 2015 onwards

**Fig. 4:**
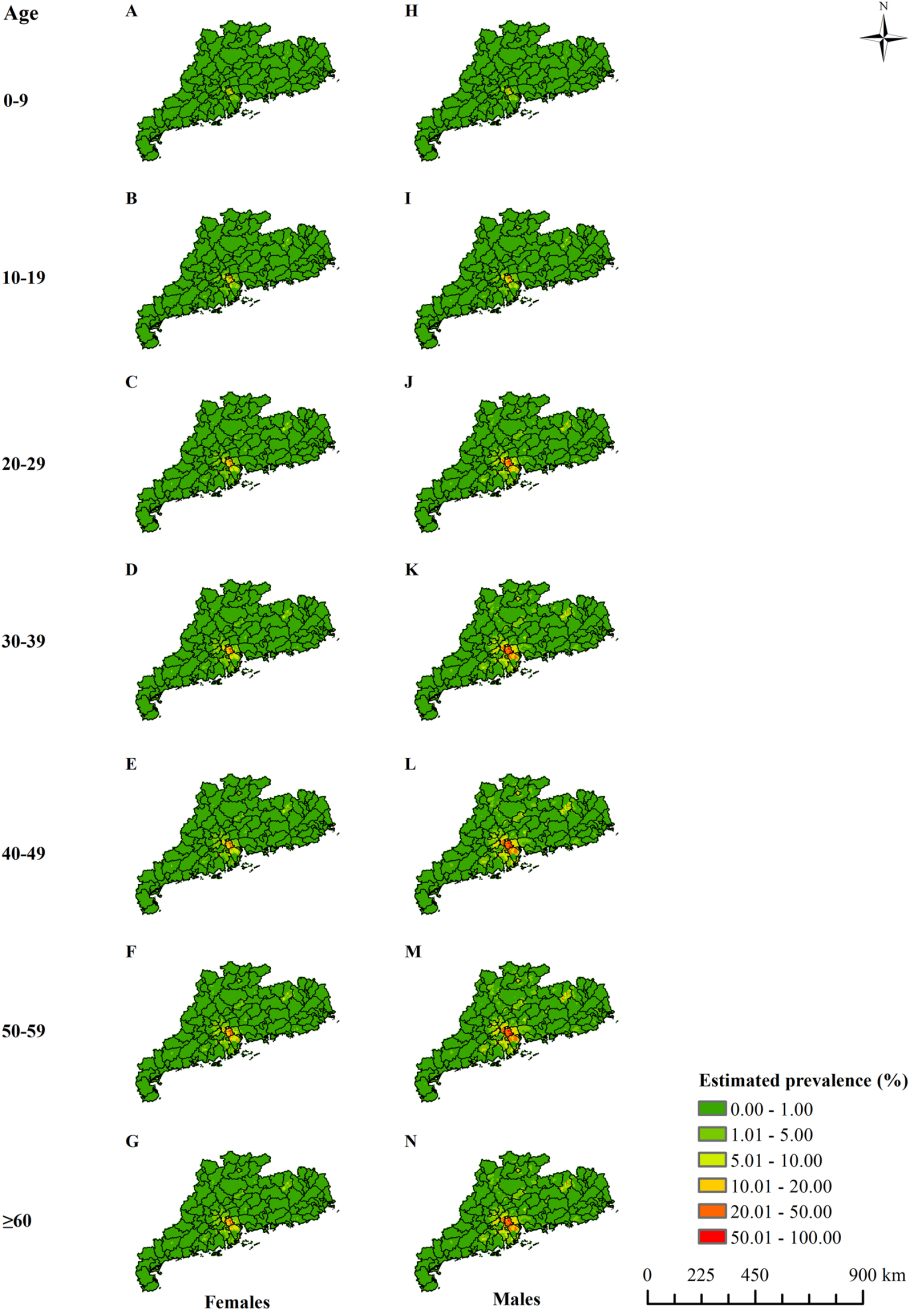
Geographical distribution of age- and gender-specific *Clonorchis* *sinensis* infection risk in Guangdong from 2015 onwards. **A**–**G** Present *C.* *sinensis* infection of males in age groups 0–9, 10–19, 20–29, 30–39, 40–49, 50–59 and ≥ 60 years old. **H**–**N** Present *C.* *sinensis* infection of females in age groups 0–9, 10–19, 20–29, 30–39, 40–49, 50–59 and ≥ 60 years old, respectively

The model-based estimated risk maps of *C.* *sinensis* infection in different periods are presented in Fig. 3. Risk maps showed that the *C.* *sinensis* infection risk increased, followed by a relatively stable period, and then gradually decreased. In the recent time period (from 2015 onwards), endemic areas were mainly distributed in the Pearl River Delta and a few areas of northeastern Guangdong. In the Pearl River Delta, high prevalence (> 20%) areas were majorly estimated in the cities of Foshan, Zhongshan, Zhuhai and Jiangmen. Particularly in some areas of southern Foshan, the estimated prevalence was > 50%. In the northeastern part, moderate infection prevalence (5–20%) existed in a few areas of Heyuan and Shaoguan City. High estimation uncertainty was mainly seen in the Pearl River Delta and some northeastern areas, particularly for estimates in the 1995–1999 and 2005–2009 periods (Additional file 1: Fig. S2). Moreover, the gender- and age-specific high-resolution risk maps for the six time periods (Fig. 4 and Additional file 1: Figs. S3–S7) showed that the infection risk of females was lower than that of males in each age group, and the infection risk increased with age until 50–59 years old and then gradually decreased.

**Fig. 5:**
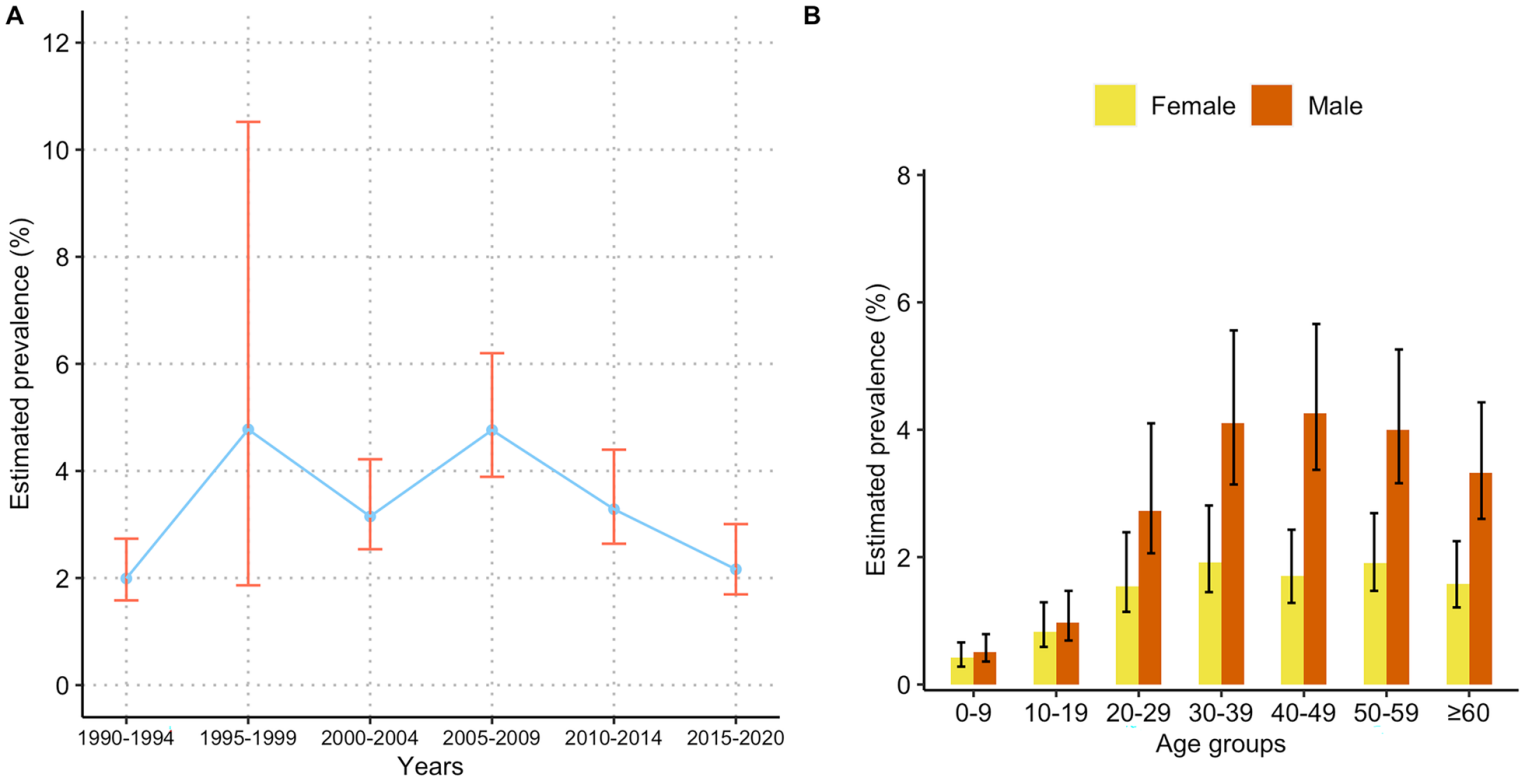
The estimated prevalence of *Clonorchis* *sinensis* infection in Guangdong. **A** Temporal trends in estimated prevalence; **B** estimated prevalence of different age-gender groups in 2015 onwards

The overall population-adjusted prevalence (Fig. 5A and Additional file 1: Table S3) showed a first increase until 1995–1999, followed by relatively stable periods until 2005–2009, and has subsequently decreased since then, a similar temporal trend as in the risk maps. In the period of 2015 onwards, the overall population-adjusted prevalence was estimated to be 2.2% (95% BCI: 1.7–3.0%), corresponding to 2.64 (95% BCI: 2.07–3.67) million infected individuals. The population-adjusted prevalence and number of infected individuals for all 21 prefecture-level cities in 2015 onwards are presented in Additional file 1: Table S4. Results indicated that Foshan, Zhongshan and Zhuhai were the top three cities with the highest prevalence of 11.2% (95% BCI: 8.4–16.3%), 7.8% (95% BCI: 4.7–16.3%) and 3.7% (95% BCI: 1.5–9.4%), respectively. On the other hand, the overall population-adjusted estimated prevalence in each age and gender group across Guangdong (Fig. 5B) demonstrated that females had a lower prevalence than males. For females, the prevalence increased with age until 30–39 years old and then became stable. For males, the prevalence rose until 40–49 years old, followed by a slow decrease in the older age groups.

## Discussion

In this study, we developed a Bayesian geostatistical model based on all available survey data since 1990 in Guangdong Province to depict age- and gender-specific high-resolution risk maps of *C.* *sinensis* infection across the province over 30 years. The outcomes reflected the spatial temporal characteristics of *C.* *sinensis* infection risk in Guangdong, providing an important reference for understanding the epidemiological status of clonorchiasis in the province. Furthermore, the added value of our study included the followings: First, we estimated the spatial temporal changes of *C.* *sinensis* infection risk and the current status in Guangdong. In the recent time period, the overall prevalence gradually decreased to 2.2%, and high risk areas shrank to Foshan, Zhongshan, Zhuhai and Jiangmen cities, which suggested the effectiveness of the control effort in Guangdong. Second, we depicted age- and gender-specific risk maps at high spatial resolution and estimated the prevalence of different age-gender groups, which present the age and gender distribution across all locations, thus guiding areas with different infection risks to choose targeted control strategies for different age and gender groups. Third, we identified several factors like precipitation, land cover and night light that were significantly related to *C.* *sinensis* infection in Guangdong.

Even though our results revealed a decreasing trend of *C.* *sinensis* infection risk in Guangdong in recent years, significant spatial heterogeneity existed across the study region. The Pearl River Delta still had a highly endemic status, and parts of northeastern Guangdong exhibited a moderate prevalence, consistent with previous studies [22]. For a long time, people living in those areas have followed the custom of eating raw or undercook fish, which could be the primary reason for higher prevalence compared to other areas. The other possible reason may be that the Pearl River Delta is an economically developed region, where people have more possibilities to obtain raw fish, a relatively expensive commodity [21]. Regarding the temporal trend, from 1990–1994 to 1995–1999, the estimated prevalence presented an increased tendency. This was probably due to increased exposure to raw fish dishes, potentially driven by the rapid economic expansion and growth of the aquaculture industry [35, 36]. From 1995–1999 to 2005–2009, the infection prevalence showed a relatively stable pattern due to the promotion of albendazole in 1993 [37] and the implementation of the “Plan for Control of Parasitic Diseases during the Ninth Five-Year Program in Guangdong Province” in 1996 [38, 39]. Towards the end of 2005, the Guangdong government introduced the “Plan for Control and Prevention of Priority Endemic Disease in Guangdong Province (2005–2010)” [40]. Subsequently, additional control schemes were issued in 2006, including the “Implementation Plan for the Control of Clonorchiasis in Guangdong Province (2006–2010)” and the “Technical Plan for the Control of Clonorchiasis in Guangdong Province (Trial)” [41]. In compliance with these policies, seven demonstrative pilots for comprehensive intervention were established [42–44], and diverse regions in the province also initiated varying degrees of control and prevention work [45]. In 2017, the Guangdong government initiated the “Plan for Prevention and Control of Key Parasitic Disease in Guangdong Province (2016–2020)” to further enhance the control of clonorchiasis [46]. All these actions may have led to a gradual downward trend in infection risk in recent years.

We found that men had a higher infection risk than women, and adults had a higher risk than children. This was most likely because men and adults were more likely to consume raw fish dishes than women and children [22]. In addition, we found several factors significantly related to *C.* *sinensis* infection in Guangdong. Environmental factors, such as precipitation and land cover, were identified as relevant factors, similar to findings on *C.* *sinensis* infection in South Korea [28]. One possible explanation was that these factors may have an effect on the survival and reproductive capabilities of intermediate hosts, subsequently influencing the risk in those specific regions. We also found that a socioeconomic factor, night light, was positively associated with infection risk, indicating the disease was more likely to occur in areas with a more developed economy. This finding aligned with prior clonorchiasis studies in China [45, 47] but differed from results of studies in Korea [28] and studies on *Opisthorchis viverrine*, another important species of foodborne parasitosis endemic in southeast Asia [27]. Notably, in our study, the distance to the nearest water body was found insignificant in relation to *C.* *sinensis* infection; thus, it was not selected after the variable selection procedure. This was inconsistent with results of Lai’s study, the study region of which was all of China [26]. As most areas of Guangdong are covered by dense river networks, there was not much difference in distances to the nearest water body across the whole region, which may lead to no significant relationship between this factor and the infection risk.

Based on the above findings, targeted measures were suggested to assist the control and prevention of *C.* *sinensis* in Guangdong. First, high population heterogeneity of infection risk demonstrated that a population-specific strategy is needed. Preventive chemotherapy should be prioritized and implemented for males and adults who frequently consume raw fish [48]. Second, mass health education is essential. Targeted health education should be provided for raising awareness about examination and treatment among males and adults [13]. For young children, who are less entrenched in their customs and dietary habits than adults [49–51], education especially in schools should focus on increasing awareness of the danger and prevention of clonorchiasis, aiming to prevent raw fish consumption. Moreover, our findings indicated that high-risk areas were concentrated in the Pearl River Delta. Hence, it is crucial for the government to further enhance the establishment of the monitoring network, especially in the Pearl River Delta. This network should cover not only the surveillance of infection rates among humans and freshwater fish but also the sanitary environment within the region. Continuous monitoring should be sustained in historically endemic areas to prevent any potential reemergence of the disease.

This study had some limitations. First, to make full use of all available survey data, we introduced an age-gender-specific variable to analyze both age- and gender-specific data together with data aggregated across age and gender. Sensitivity analysis indicated that there was no significant difference between the overall population-adjusted estimated prevalence calculated based on age- and gender-specific prevalence and that based on surveys aggregated across age and gender, suggesting the method was rational. Second, we did not consider some key relevant covariates in the model, such as the distribution of residents’ dietary habit of eating raw fish, distribution of freshwater snails (e.g. *Parafossarulus striatulus*) and information on the detailed implementation of local control measures in different areas of Guangdong because of the unavailability of these factors. Nevertheless, the model validation showed a reasonable capacity for estimation accuracy. Third, discretizing continuous variables might lead to some problems, such as information loss and spurious effects. In our study, we converted continuous variables to multiple categories based on previous research and preliminary exploration to avoid the above problems as much as possible. Furthermore, we discretized continuous variables for the following reasons: (i) we discretized continuous variables to capture the potential non-linear outcome-predictor relationships; (ii) for certain variables, such as age, the categorical form can offer easier epidemiological interpretation, which is helpful for policymakers to understand and apply as they put control strategies in place; (iii) considering privacy protection, the data we obtained was grouped by gender and age; (iv) there were few survey data for certain years, so we divided the whole study period into segments to depict temporal variations rather than analyzing yearly infection risk. To avoid more complex models and high estimation uncertainty, we divided the period into 5-year categories, which also fits the control promotions in Guangdong. In addition, data from 2015 onwards were not further divided into finer time intervals, given that most survey data were obtained in the years up to 2019, with fewer data available for the subsequent years. Results can be updated in the future if further representative and comparable surveys are obtained in subsequent years.

## Conclusions

In conclusion, we present high-resolution model-based estimates of *C.* *sinensis* infection in Guangdong Province, China, identifying spatial, temporal, age and gender heterogeneities, thus providing useful information for control and prevention of the disease in the province.

### Supplementary Information

**Additional file 1: Table S1**. Environmental, socioeconomic and demographic data sources. **Table S2.** Overview of characteristics of clonorchiasis survey data in Guangdong Province. **Figure S1.** Results of model validation. **Figure S2.** Estimation uncertainty in Guangdong Province in different time periods. **Figure S3.** Geographical distribution of age- and gender-specific *Clonorchis* *sinensis* infection risk in Guangdong, 1990–1994. **Figure S4.** Geographical distribution of age- and gender-specific *Clonorchis sinensis* infection risk in Guangdong, 1995–1999. **Figure S5.** Geographical distribution of age- and gender-specific *Clonorchis sinensis* infection risk in Guangdong, 2000–2004. **Figure S6.** Geographical distribution of age- and gender-specific *Clonorchis sinensis* infection risk in Guangdong, 2005–2009. **Figure S7.** Geographical distribution of age- and gender-specific *Clonorchis sinensis* infection risk in Guangdong, 2010–2014. **Table S3.** Age- and gender-adjusted estimated prevalence (%) and the number of individuals (× 10^3^) infected with *Clonorchis* *sinensis* in Guangdong Province. **Table S4.** Age- and gender-adjusted estimated prevalence (%) and number of individuals (× 10^3^) infected with *Clonorchis* *sinensis* in Guangdong Province, stratified by city in 2015 onwards.

## Data Availability

The data generated from the literature are available online. The data from large-scale surveys cannot be shared publicly because of the confidentiality required by the Guangdong Provincial Center for Disease Control and Prevention. Researchers can contact the Guangdong Provincial Center for Disease Control and Prevention at + 86-020-31051692 or sjkzxjfs@gd.gov.cn to apply for these data. All other data are available from the open-access databases.

## Abbreviations

*C. sinensis*: *Clonorchis sinensis*
IARC: International Agency for Research on Cancer
Guangdong CDC: Guangdong Provincial Center for Disease Control and Prevention
CNKI: China National Knowledge Internet
LST: Land surface temperature
NDVI: Normalized difference vegetation index
INLA: Integrated nested Laplace approximations
ME: Mean error
MAE: Mean absolute error
BCI: Bayesian credible interval
ROC: Receiver operating characteristic
AUC: Area under curve
