# Supplementary material for: High-resolution mapping of age- and gender-specific risk of Clonorchis sinensis infection risk in Guangdong, China: a geostatistical modeling study
Source: Parasit Vectors. 2024 Feb 16;17:67. doi: 10.1186/s13071-024-06166-z (PMC10873974; doi:10.1186/s13071-024-06166-z)
Supplement: Supplementary file 1 — Additional file 1: Table S1. Environmental, socioeconomic and demographic data sources. Table S2. Overview of characteristics of clonorchiasis survey data in Guangdong Province. Figure S1. Results of model validation. Figure S2. Estimation uncertainty in Guangdong Province in different time periods. Figure S3. Geographical distribution of age- and gender-specific Clonorchis sinensis infection risk in Guangdong, 1990–1994. Figure S4. Geographical distribution of age- and gender-specific Clonorchis sinensis infection risk in Guangdong, 1995–1999. Figure S5. Geographical distribution of age- and gender-specific Clonorchis sinensis infection risk in Guangdong, 2000–2004. Figure S6. Geographical distribution of age- and gender-specific Clonorchis sinensis infection risk in Guangdong, 2005–2009. Figure S7. Geographical distribution of age- and gender-specific Clonorchis sinensis infection risk in Guangdong, 2010–2014. Table S3. Age- and gender-adjusted estimated prevalence (%) and the number of individuals (× 103) infected with Clonorchis sinensis in Guangdong Province. Table S4. Age- and gender-adjusted estimated prevalence (%) and number of individuals (× 103) infected with Clonorchis sinensis in Guangdong Province, stratified by city in 2015 onwards. [file 13071_2024_6166_MOESM1_ESM.docx]

Table S1. Environmental, socioeconomic, and demographic data sources^a^.

| Sources | Data Type | Temporal coverage | Temporal resolution | Spatial resolution |
| --- | --- | --- | --- | --- |
| MODIS/Terra^b^ | LST^j^ in the daytime | 2000-2020 | 8 days | 1 km |
|  | LST^j^ at night | 2000-2020 | 8 days | 1 km |
|  | NDVI^k^ | 2000-2020 | 16 days | 1 km |
|  | Land cover | 2001-2020 | Yearly | 1 km |
| resdc^c^ | Precipitation | 2000-2015 | Yearly | 1 km |
|  | GDP per capita | 1995; 2005; 2010; 2015; 2019 | Yearly | 1 km |
| SEDAC^d^ | Urban extents | 1995 | Yearly | 1 km |
|  | HII^l^ | 1995-2004 | Yearly | 1 km |
| SRTM^e^ | Elevation | 2000 | Yearly | 1 km |
| NCEI^f^ | Nightlight | 1992-2018 | Yearly | 1 km |
| PSL^g^ | Soilmoisture | 1990-2020 | Monthly | 60 km |
| SWBD^h^ | Water bodies | 2000 | Yearly | - |
| WorldPop^i^ | Population data | 2000; 2005; 2015 | Yearly | 1 km |

^a^Data accessed in May 2022

^b^Moderate Resolution Imaging Spectroradiometer (MODIS)/Terra; available at: <http://modis.gsfc.nasa.gov/>.

^c^Resource and Environment Science and Data Center (resdc); available at: <https://www.resdc.cn/>.

^d^Socioeconomic Data and Applications Center (SEDAC); available at: <http://sedac.ciesin.org/>.

^e^Shuttle Radar Topography Mission (SRTM); available at: <https://www2.jpl.nasa.gov/srtm/>.

^f^National Centers for Environmental Information (NCEI); available at: <https://www.ngdc.noaa.gov/ngdc.html/>.

^g^Physical Sciences Laboratory (PSL); available at: https://psl.noaa.gov/.

^h^Shuttle Radar Topography Mission Water Body Data (SWBD), available at: http://gis.ess.washington.edu/data/vector/worldshore/index.html/.

^i^The WorldPop project; available at: <http://www.worldpop.org.uk/>.

^j^Land surface temperature (LST).

^k^Normalized difference vegetation index (NDVI).

^l^Human influence index (HII).

Table S2. Overview of characteristics of clonorchiasis survey data in Guangdong Province.

| Survey year | | | | | 1990-1994 | 1995-1999 | 2000-2004 | 2005-2009 | 2010-2014 | ≥2015 | Total |
| --- | --- | --- | --- | --- | --- | --- | --- | --- | --- | --- | --- |
| No. of surveys/ locations | | | | | 121/120 | 8/8 | 100/84 | 62/61 | 161/125 | 154/112 | 606/463 |
|  | | Literatures | | | 1/1 | 8/8 | 49/43 | 62/61 | 127/91 | 83/46 | 330/211 |
|  | | Large-scale surveys | | | 120/119 | - | 51/49 | - | 34/34 | 71/66 | 276/246 |
| No. of individuals | | | | | 62,141 | 6,430 | 71,218 | 50,369 | 58,317 | 35,540 | 284,015 |
|  | | Literatures | | | 624 | 6,430 | 44,859 | 50,369 | 43,736 | 23,139 | 169,157 |
|  | | Large-scale surveys | | | 61,517 | - | 26,359 | - | 14,581 | 12,401 | 114,858 |
|  |  | | Gender | |  |  |  |  |  |  |  |
|  |  | |  | Female | 32,513 | - | 13,237 | - | 7,164 | 6,236 | 59,150 |
|  |  | |  | Male | 29,004 | - | 13,122 | - | 7,417 | 6,165 | 55,708 |
|  |  | | Age (years) | |  |  |  |  |  |  |  |
|  |  | |  | < 10 | 15,674 | - | 5,128 | - | 2,069 | 2,264 | 25,135 |
|  |  | |  | 10-19 | 11,926 | - | 5,685 | - | 2,683 | 1,704 | 21,998 |
|  |  | |  | 20-29 | 8,780 | - | 2,636 | - | 1,658 | 1,190 | 14,264 |
|  |  | |  | 30-39 | 9,953 | - | 4,465 | - | 2,000 | 1,831 | 18,249 |
|  |  | |  | 40-49 | 5,809 | - | 3,587 | - | 2,276 | 1,858 | 13,530 |
|  |  | |  | 50-59 | 4,683 | - | 2,539 | - | 1,987 | 1,656 | 10,865 |
|  |  | |  | ≥ 60 | 4,692 | - | 2,319 | - | 1,908 | 1,898 | 10,817 |
| Raw prevalence (%) | | | | | 1.82 | 5.21 | 13.33 | 19.04 | 10.24 | 8.37 | 10.38 |

**
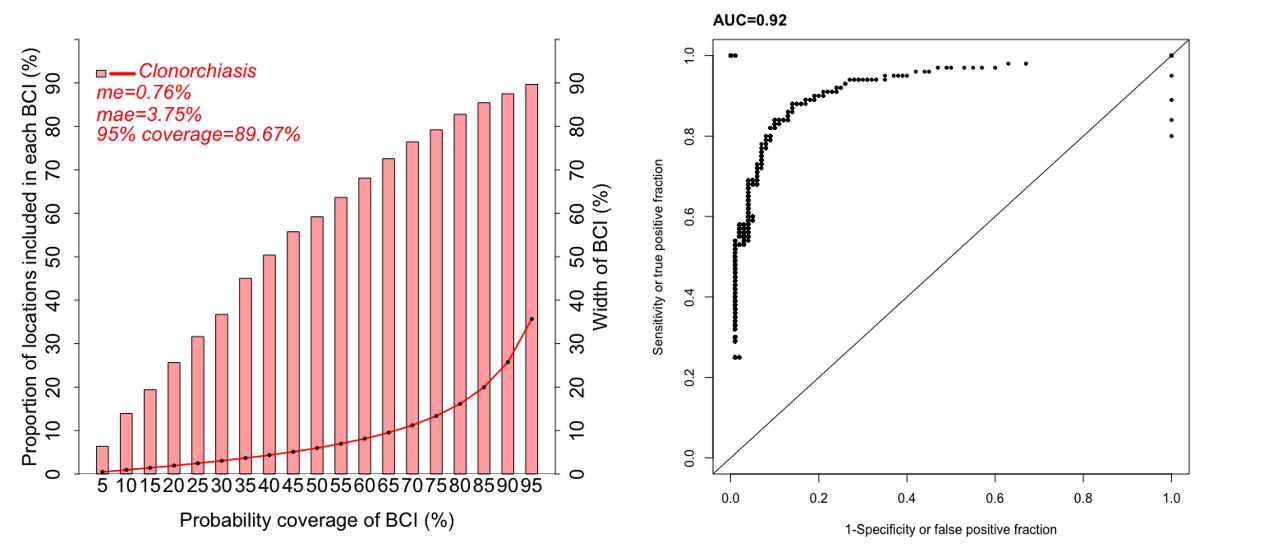
**

**Fig. S1.** The results of model validation.


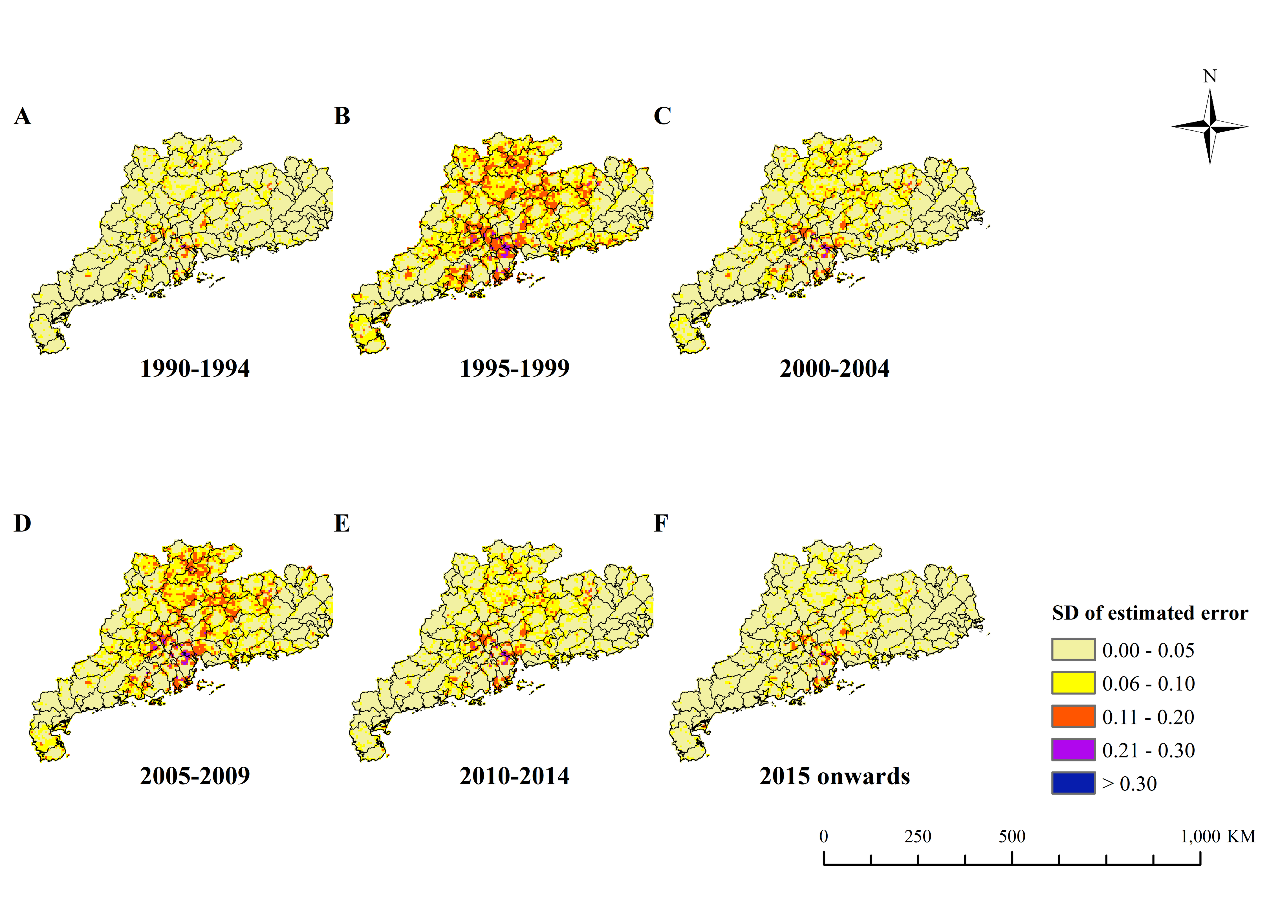


**Fig. S2.** The estimation uncertainty in Guangdong Province in different time periods.


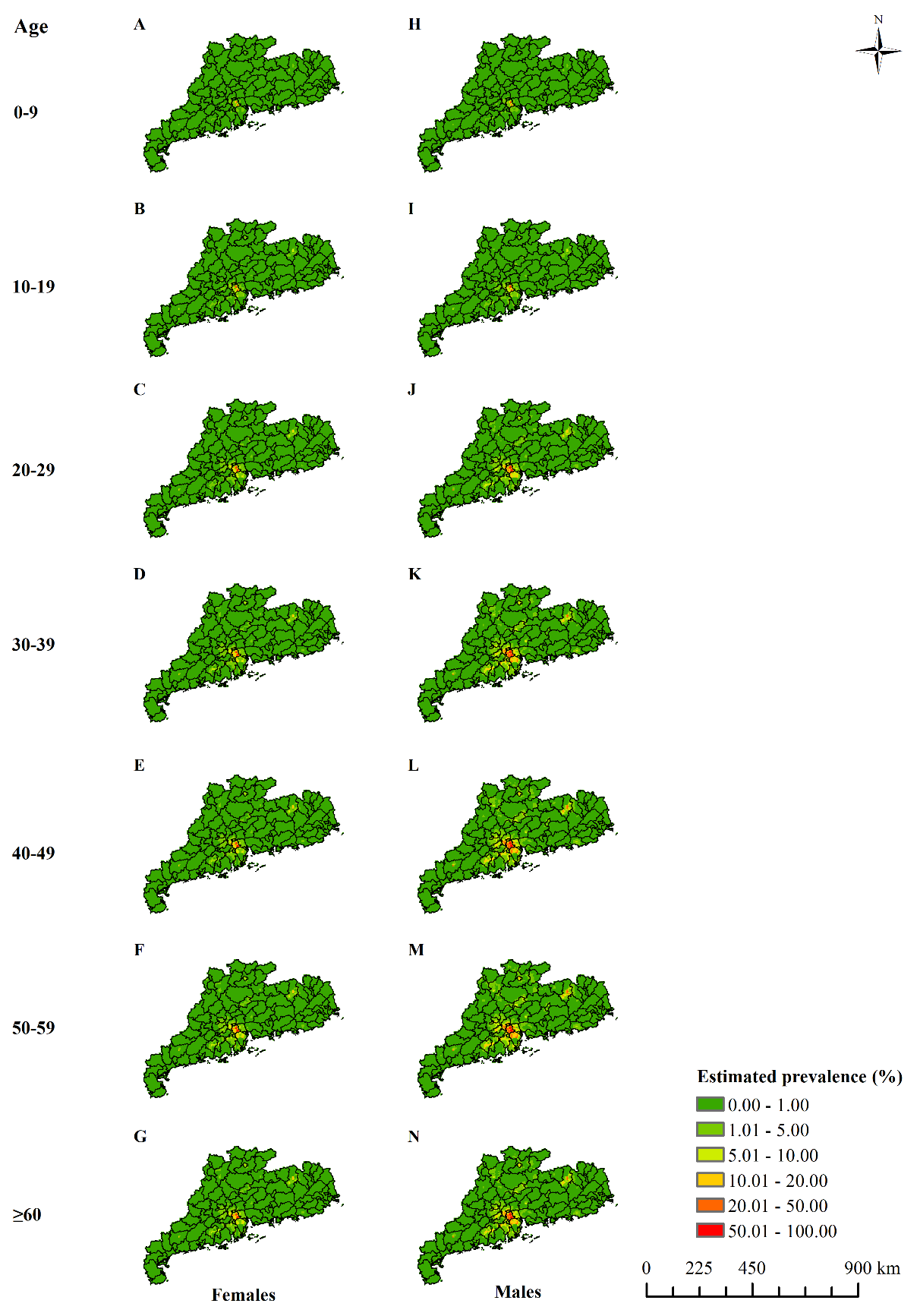


**Fig. S3.** The geographical distribution of age- and gender specific *C. sinensis* infection risk in Guangdong, 1990-1994.

**
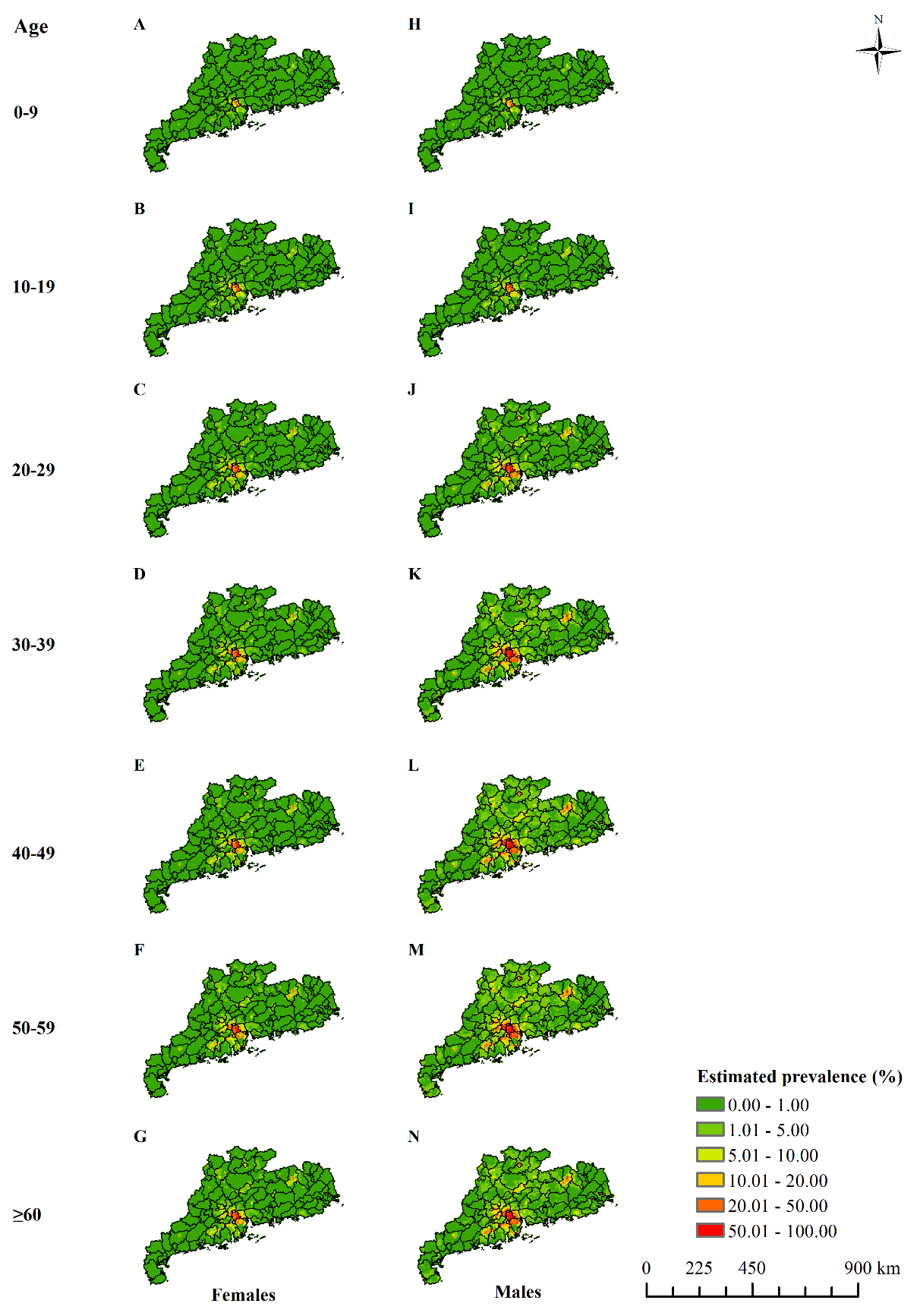
**

**Fig. S4.** The geographical distribution of age- and gender specific *C. sinensis* infection risk in Guangdong, 1995-1999.

**
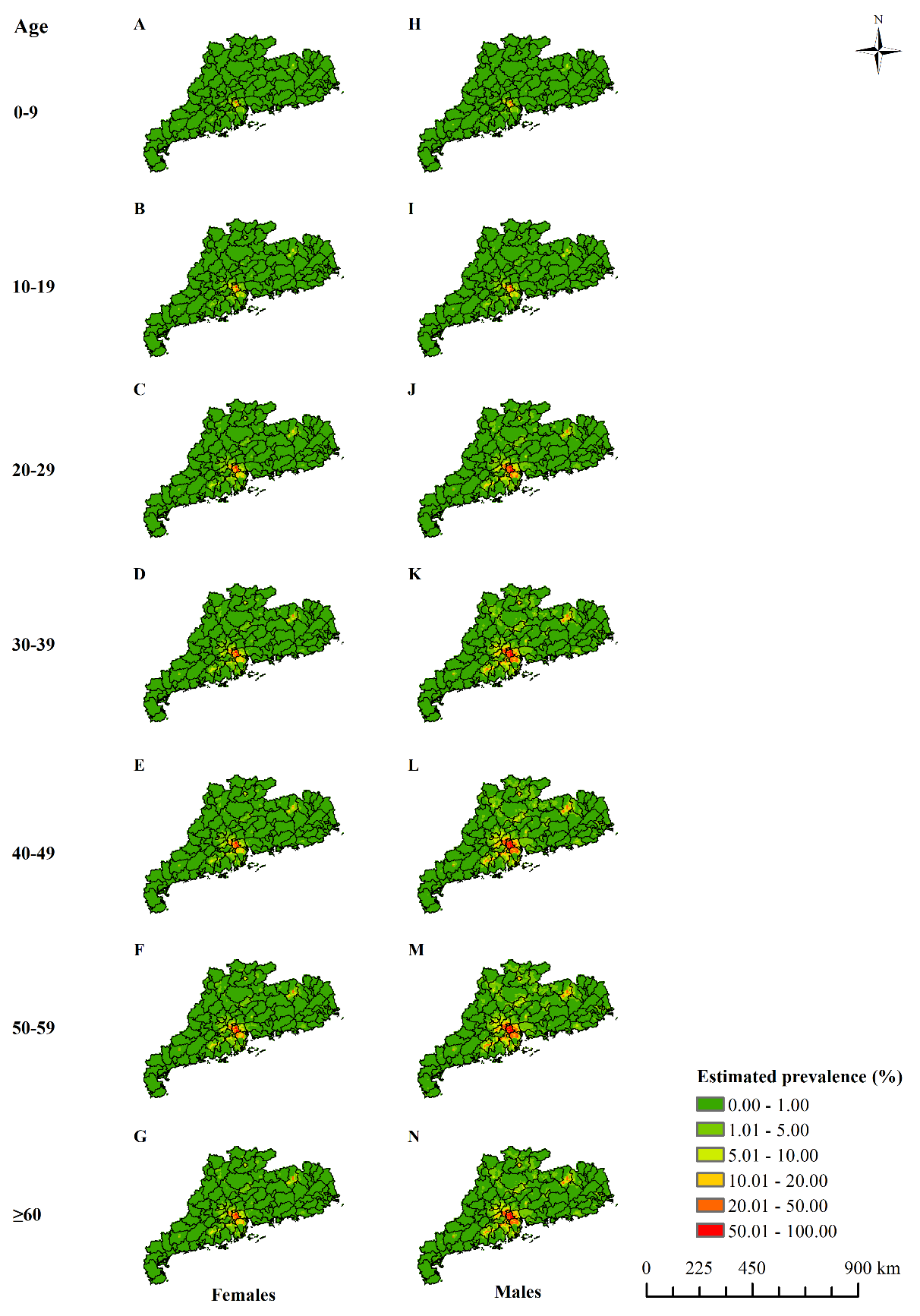
**

**Fig. S5.** The geographical distribution of age- and gender specific *C. sinensis* infection risk in Guangdong, 2000-2004.

**
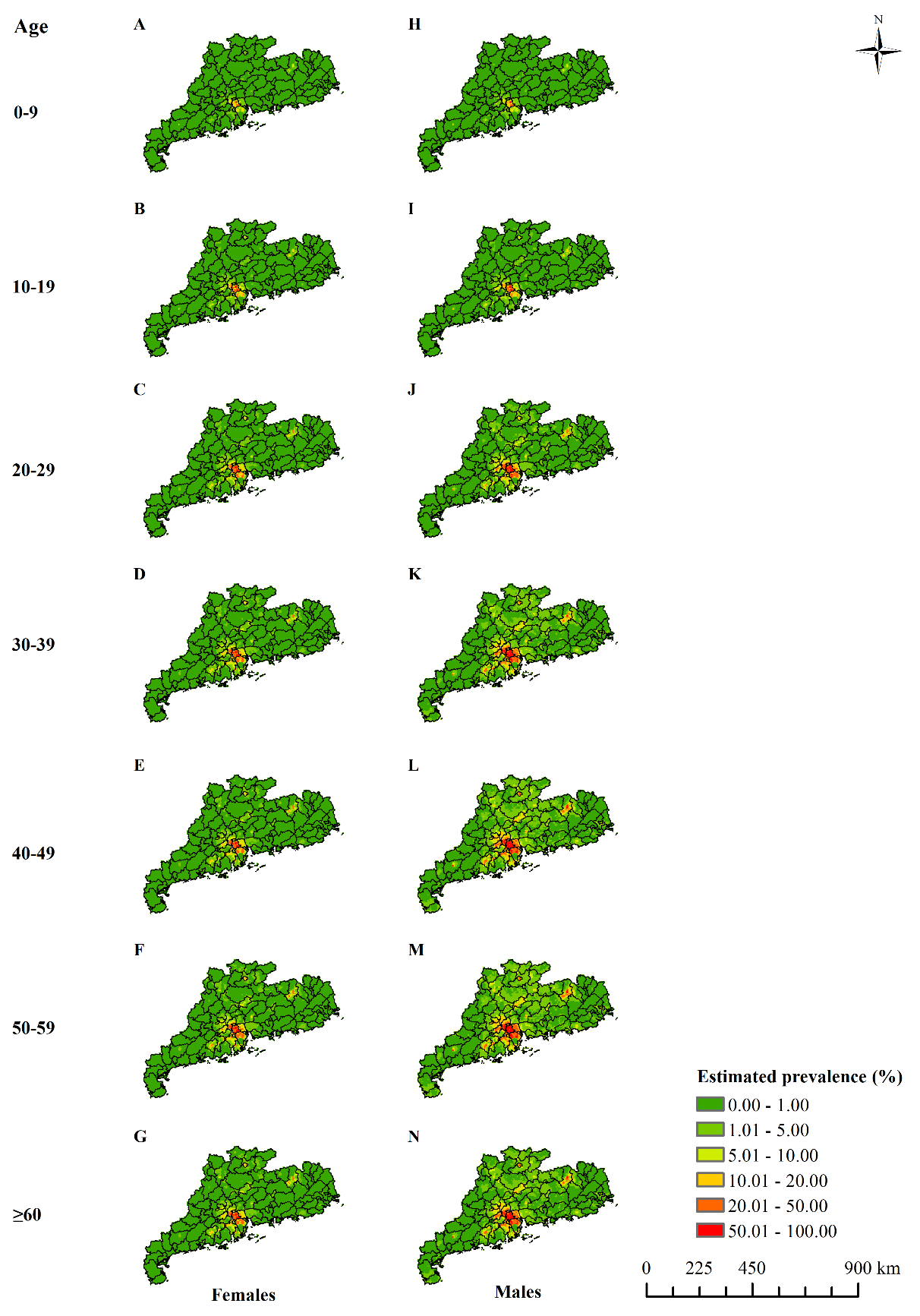
**

**Fig. S6.** The geographical distribution of age- and gender specific *C. sinensis* infection risk in Guangdong, 2005-2009.

**
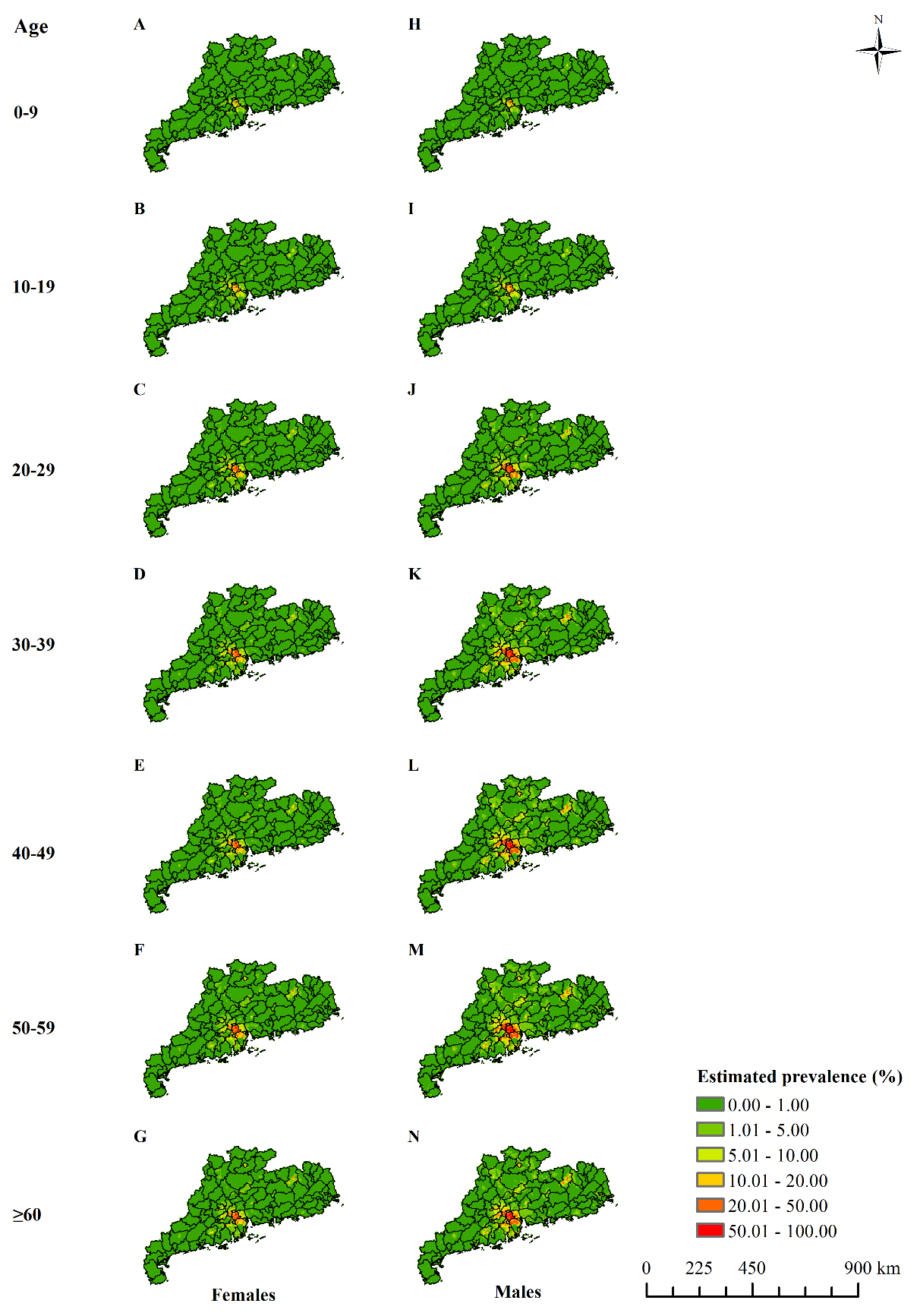
**

**Fig. S7.** The geographical distribution of age- and gender specific *C. sinensis* infection risk in Guangdong, 2010-2014.

Table S3. Age- and gender- adjusted estimated prevalence (%) and number of individuals (×10^3^) infected with *C. sinensis* in Guangdong Province.

| Years | Estimated prevalence (%) | No. infected (×10^3^) |
| --- | --- | --- |
| 1990-1994 | 1.99 (1.58, 2.73) | 1343.77 (1067.67, 1844.48) |
| 1995-1999 | 4.78 (1.86, 10.52) | 3719.59 (1451.78, 8194.12) |
| 2000-2004 | 3.15 (2.54, 4.22) | 2790.78 (2247.94, 3737.80) |
| 2005-2009 | 4.76 (3.89, 6.20) | 4602.95 (3759.16, 5991.56) |
| 2010-2014 | 3.29 (2.64, 4.40) | 3613.47 (2904.19, 4836.66) |
| 2015 onwards | 2.16 (1.69, 3.01) | 2638.16 (2067.47, 3668.40) |

Table S4. Age- and gender- adjusted estimated prevalence (%) and number of individuals (×10^3^) infected with *C. sinensis* in Guangdong Province, stratified by city from 2015 onwards.

| Cities | Estimated prevalence (%) | No. infected (×10^3^) |
| --- | --- | --- |
| Foshan | 11.22 (8.42, 16.32) | 901.89 (676.66, 1312.34) |
| Zhongshan | 7.78 (4.71, 16.25) | 283.58 (171.88, 592.47) |
| Zhuhai | 3.70 (1.54, 9.42) | 62.24 (25.96, 158.62) |
| Jiangmen | 2.78 (1.78, 5.85) | 143 (91.75, 301.16) |
| Shaoguan | 2.10 (0.90, 5.22) | 68.01 (29.17, 169.48) |
| Guangzhou | 1.85 (1.13, 3.73) | 269.18 (163.49, 541.94) |
| Dongguan | 1.61 (0.29, 8.82) | 153.20 (27.32, 837.58) |
| Zhaoqing | 1.46 (0.71, 3.28) | 66.43 (32.15, 149.10) |
| Heyuan | 1.37 (0.71, 3.26) | 47.72 (24.61, 113.42) |
| Qingyuan | 1.17 (0.58, 2.68) | 51.32 (25.49, 117.25) |
| Shanwei | 0.86 (0.15, 6.58) | 29.39 (4.94, 223.51) |
| Huizhou | 0.84 (0.17, 5.47) | 47.22 (9.54, 306.16) |
| Yunfu | 0.63 (0.11, 3.80) | 17.18 (2.97, 104.27) |
| Chaozhou | 0.51 (0.19, 2.03) | 15.42 (5.61, 60.95) |
| Shenzhen | 0.48 (0.26, 1.36) | 61.19 (32.98, 174.52) |
| Yangjiang | 0.42 (0.07, 4.67) | 11.78 (2.02, 130.01) |
| Meizhou | 0.41 (0.17, 1.16) | 20.41 (8.15, 56.98) |
| Zhanjiang | 0.41 (0.11, 1.64) | 33.65 (8.94, 134.16) |
| Shantou | 0.40 (0.06, 3.32) | 24.75 (3.93, 204.31) |
| Maoming | 0.27 (0.09, 0.97) | 17.90 (5.85, 65.19) |
| Jieyang | 0.23 (0.09, 1.20) | 15.74 (6.10, 82.01) |
